# A Unique Presentation of Bilateral Posterolateral Corner Injury With Right Posterior Cruciate Ligament and Left Anterior Cruciate Ligament Tears

**DOI:** 10.7759/cureus.106046

**Published:** 2026-03-28

**Authors:** Pratik Shahare, Samir Dwidmuthe, Deepanjan Das, Harshavardhan Reddy

**Affiliations:** 1 Orthopedics, All India Institute of Medical Sciences, Nagpur, Nagpur, IND

**Keywords:** bilateral knee injury, cruciate ligament tear, ligament reconstruction, multiligamentous knee injury, posterolateral corner injury

## Abstract

Bilateral posterolateral corner (PLC) injuries of the knees are exceptionally rare, particularly when associated with asymmetric cruciate ligament involvement. This case report presents a 23-year-old man who sustained simultaneous bilateral PLC injuries with a right posterior cruciate ligament (PCL) tear and left anterior cruciate ligament (ACL) tear following a single trauma, during a stampede. Initial examination revealed bilateral varus instability with positive dial tests. Magnetic resonance imaging (MRI) confirmed combined PLC and cruciate ligament injuries with associated fibular collateral ligament (FCL) avulsion and popliteus tendon (PLT) tears bilaterally, as well as a lateral meniscus root tear on the right knee.

Surgical management was performed sequentially. The right knee underwent open PLC avulsion repair using suture anchors and augmented with an ipsilateral semitendinosus graft (Larson procedure), followed by delayed arthroscopic PCL reconstruction with a peroneus longus graft and lateral meniscus root repair. The left knee was treated with single-stage arthroscopic ACL reconstruction using a quadruple semitendinosus graft, combined with PLC repair augmented using the gracilis tendon and the repair of the avulsed biceps femoris tendon. Postoperative rehabilitation involved staged progression from immobilization to the full range of motion and weight-bearing at 8-12 weeks.

At one-year follow-up, the patient achieved right knee motion from 0° to 120° and left knee motion from 0° to 140°, with full ambulation and minimal residual laxity (grade 1 posterior drawer on the right; grade 1a Lachman on the left). This case highlights the clinical importance of the early diagnosis and selective augmented repair of avulsion-type PLC injuries. When combined with timely cruciate ligament reconstruction, such an approach may yield outcomes comparable to formal reconstruction while preserving native tissue and reducing postoperative morbidity in acute multiligamentous knee injuries.

## Introduction

The posterolateral corner (PLC) of the knee, often referred to as the "dark side of the knee" due to its diagnostic complexity, is a critical stabilizing unit consisting primarily of the fibular collateral ligament (FCL), the popliteus tendon (PLT), and the popliteofibular ligament (PFL) [1].

These structures function synergistically to resist varus angulation and external tibial rotation. While isolated PLC injuries occur, they are relatively rare; however, combined injuries are frequently encountered, accounting for up to 16% of all knee ligament injuries, most commonly in association with anterior cruciate ligament (ACL) or posterior cruciate ligament (PCL) tears [2,3].

Diagnosing PLC injuries in the acute setting remains a significant clinical challenge. The complex anatomy, combined with patient guarding, soft tissue swelling, and hemarthrosis, often obscures physical examination findings. Consequently, PLC injuries are frequently overlooked or misdiagnosed, particularly when overshadowed by more obvious cruciate ligament disruptions [4]. This diagnostic gap is critical, as unrecognized PLC insufficiency significantly increases the force on cruciate reconstruction grafts, leading to chronic instability, the failure of ACL or PCL reconstructions, and early osteoarthritis [5]. Furthermore, routine magnetic resonance imaging (MRI) may occasionally underestimate the severity of injury, especially in cases of multi-ligament involvement where anatomical landmarks are disrupted.

While unilateral multi-ligament knee injuries (MLKIs) are well-documented, simultaneous bilateral MLKIs are exceedingly rare, typically resulting from high-velocity trauma such as road traffic accidents or falls from heights. Bilateral knee dislocations or multi-ligament injuries represent a tiny fraction of orthopedic trauma, and specific patterns involving bilateral PLC disruption combined with contralateral cruciate tears have rarely been reported in the literature [6].

We present a rare case of a 23-year-old man who sustained simultaneous bilateral PLC injuries combined with a right PCL tear (with lateral meniscus root tear) and a left ACL tear following a single traumatic event. This case report highlights the diagnostic challenges, surgical decision-making, and rehabilitation strategies required for such a complex, bilateral presentation.

## Case presentation

While most PLC and ligament injuries are unilateral and result from direct sports-related impact, this case presents a unique mechanism of injury that involved a 23-year-old man having a primary fall, followed by an immediate secondary trauma in which the patient was stepped on by multiple individuals in a stampede. He presented with marked bilateral swelling, ecchymosis over the posterolateral aspects, severe pain, and restricted motion. Physical examination revealed grade 3 varus laxity in both knees. The left knee had a positive Lachman test (grade 3), while the right demonstrated a grade 3 posterior drawer. Peripheral pulses and neurological status were intact. The dial test was positive at 30° and 90° on the right (indicating PCL and PLC injury) and only at 30° on the left (suggesting isolated PLC injury). However, the bilateral involvement reduced the contralateral side's reliability for comparison.

**Figure 1:**
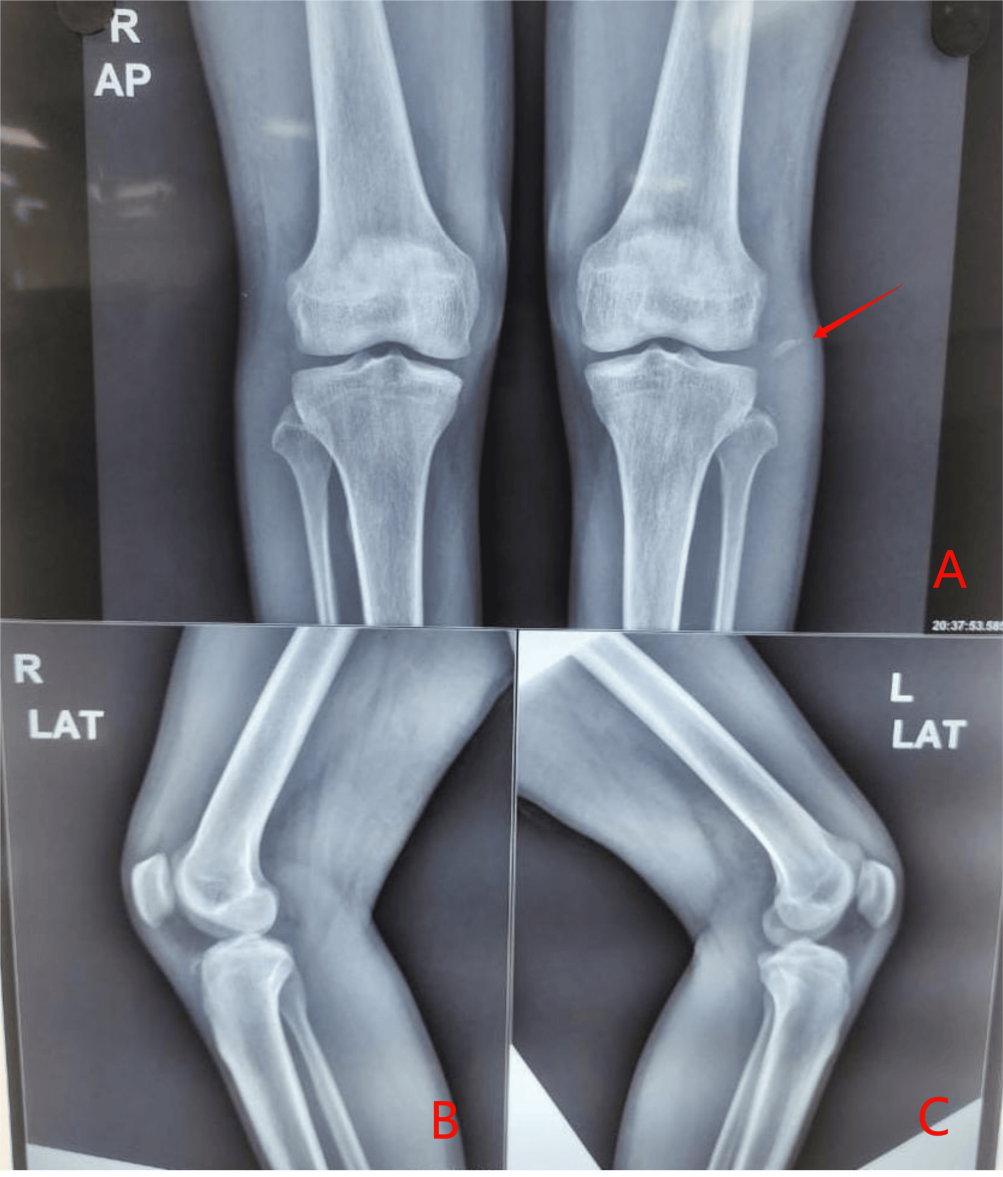
Preoperative X-ray (antero-posterior and lateral views of both knees) (A) Antero-posterior view of both knees with the arrow showing fibular head avulsion fracture in the left knee. (B and C) Lateral view of both the right and left knee

**Figure 2:**
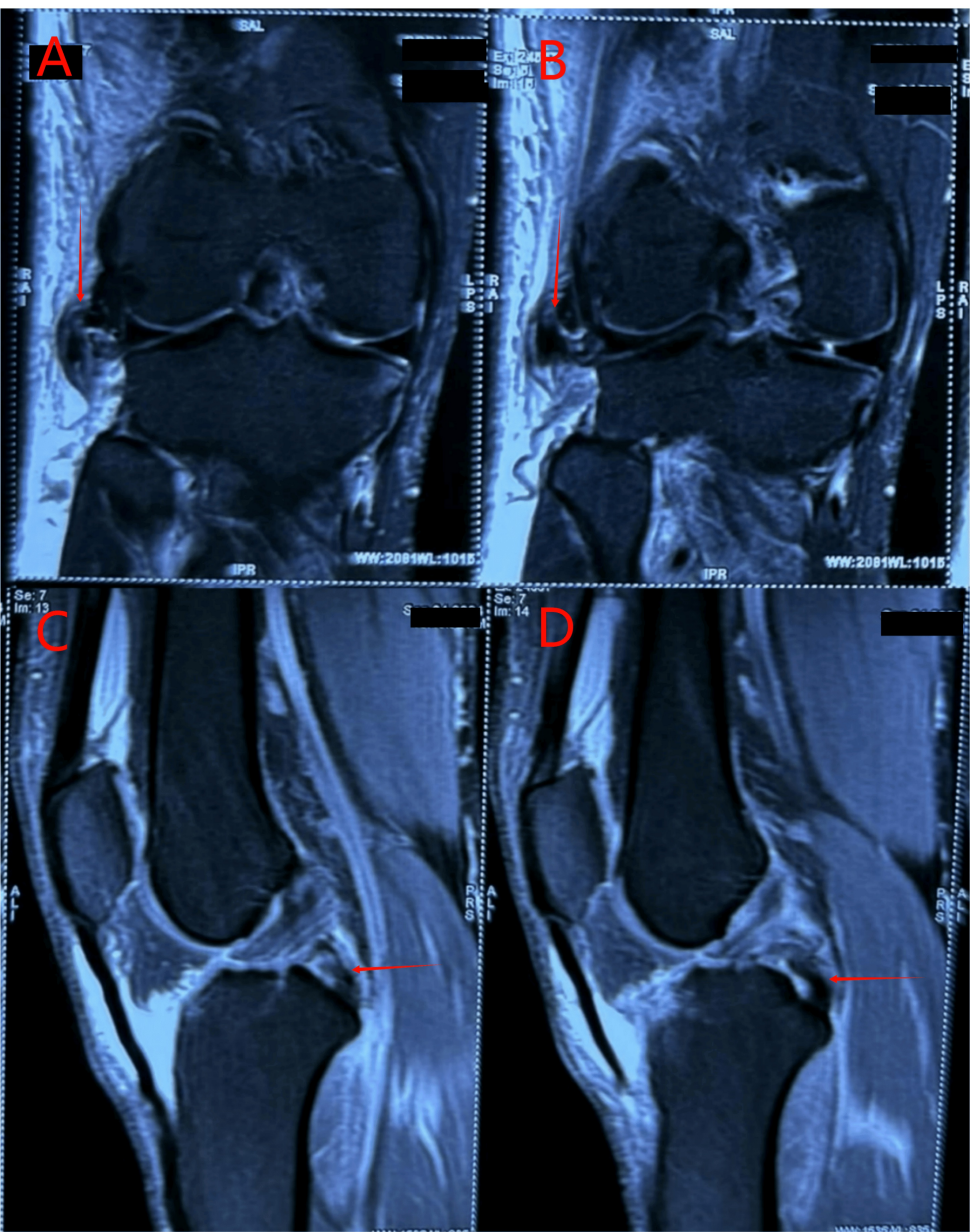
MRI of right knee (two coronal and two sagittal views) (A and B) PLC avulsion injury showing the lack of continuity in the fibers of the lateral collateral ligament in coronal views. (C and D) PCL tear showing the lack of continuity of PCL fibers in sagittal views MRI, magnetic resonance imaging; PLC, posterolateral corner; PCL, posterior cruciate ligament

**Figure 3:**
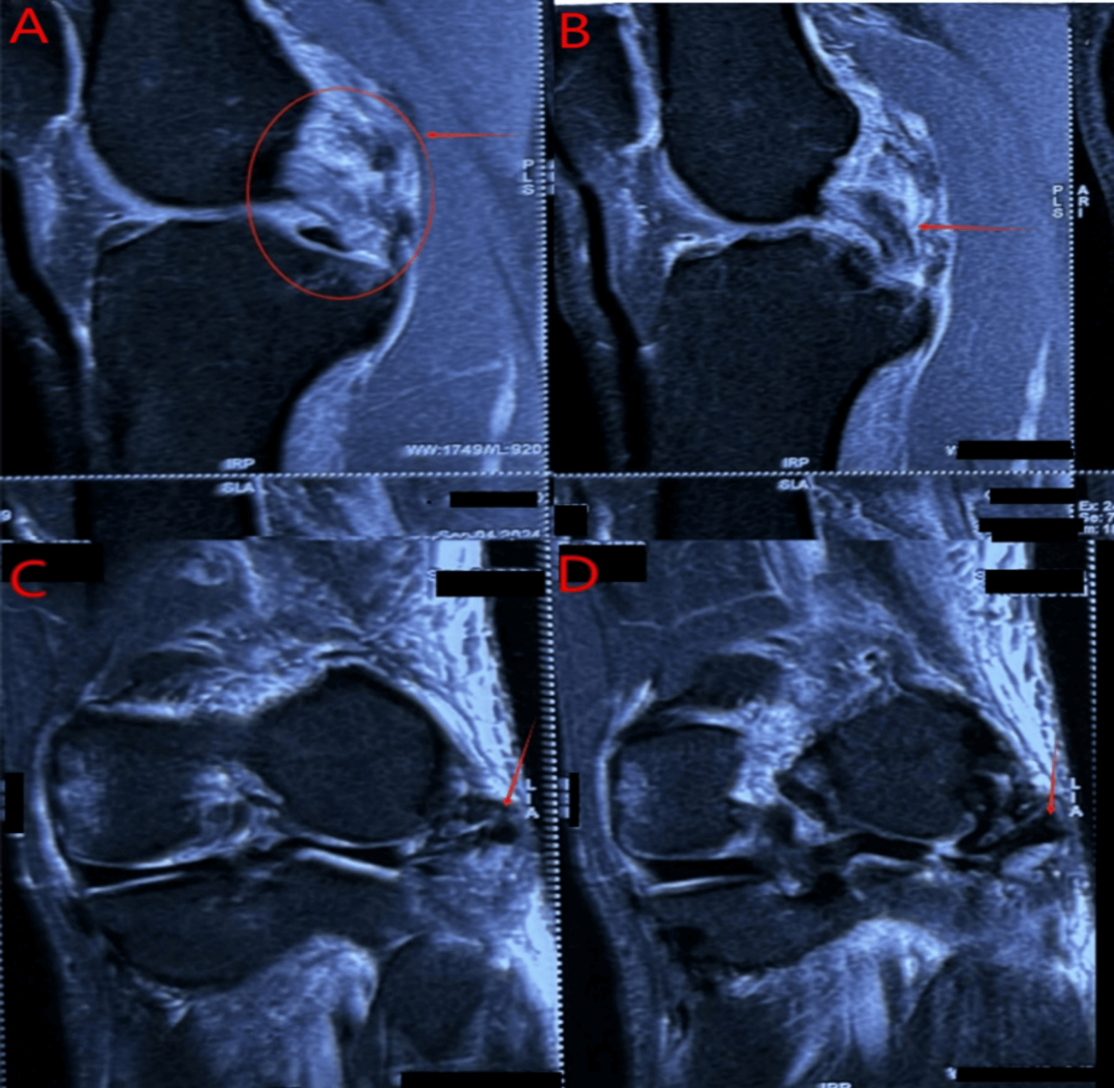
MRI of left knee showing ACL tear and PLC avulsion injury (two sagittal and two coronal views) (A) Sagittal view showing the area at the intercondylar notch (red circle with arrow) with the absence of ACL fibers. (B) Sagittal view of the intercondylar notch showing the buckling of PCL (indirect sign of ACL tear). (C) Coronal view showing PLC avulsion injury (the lack of continuous fibers of the lateral collateral ligament). (D) Coronal view showing PLC avulsion injury with avulsed lateral collateral ligament MRI, magnetic resonance imaging; PLC, posterolateral corner; PCL, posterior cruciate ligament; ACL, anterior cruciate ligament

Radiographic evaluation showed a fibular head avulsion on the left and a slight bilateral subluxation of tibiofemoral joints (Figure 1). MRI confirmed, on the right side, a complete tear of the fibular collateral ligament (FCL), popliteus tendon, and biceps femoris tendon, along with a high-grade posterior cruciate ligament (PCL) tear (Figure 2). The left knee MRI revealed a complete disruption of the FCL, avulsion from the fibular head, arcuate complex injury, complete popliteus tendon tear, and full-thickness anterior cruciate ligament (ACL) rupture (Figure 3).

**Figure 4:**
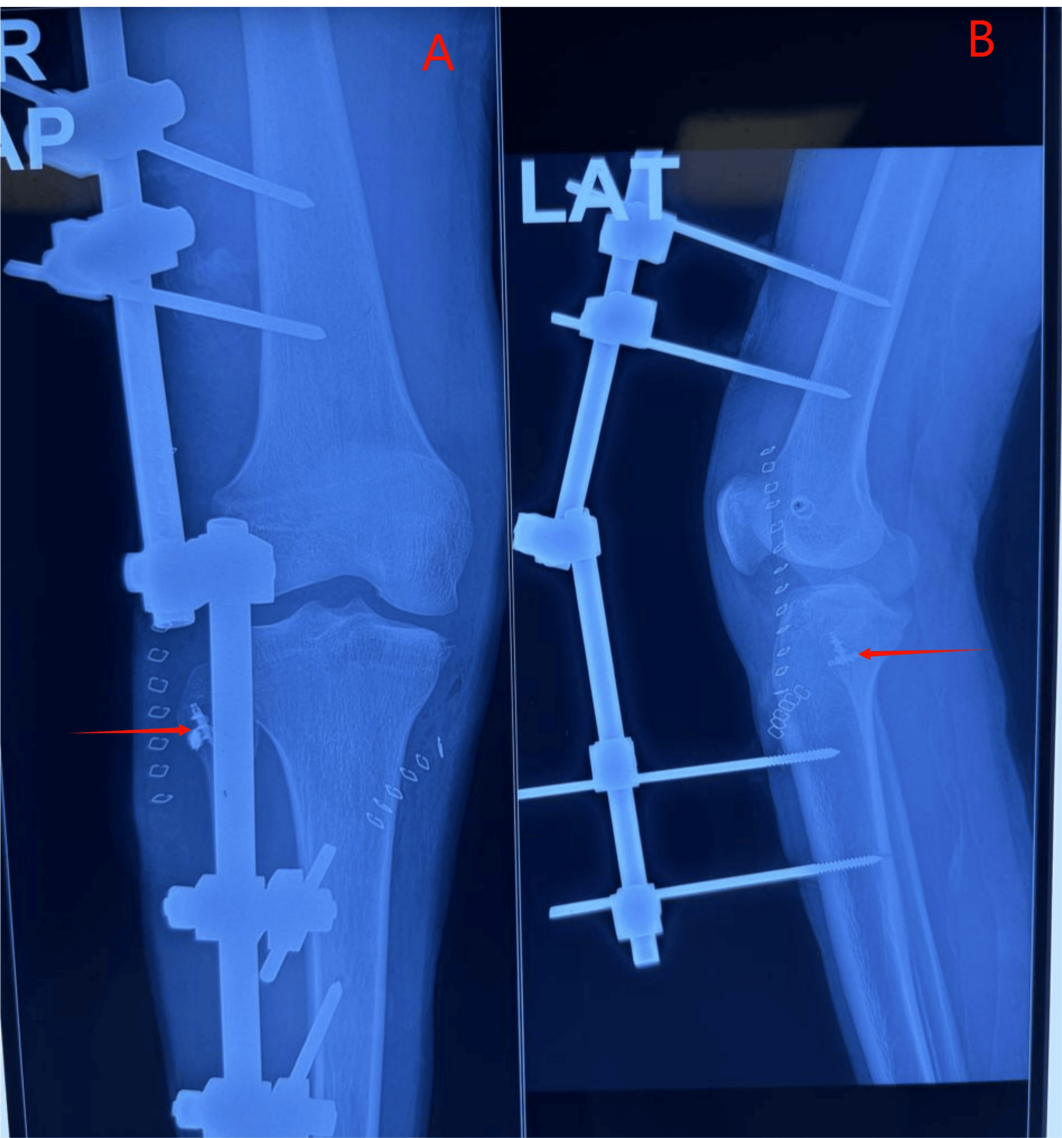
Immediate postoperative right knee X-ray (after PLC repair + Larson augmentation + external fixator application) (A) AP view with suture anchor visible in the fibular head. (B) Lateral view with suture anchor visible in the fibular head AP, antero-posterior; PLC, posterolateral corner

**Figure 5:**
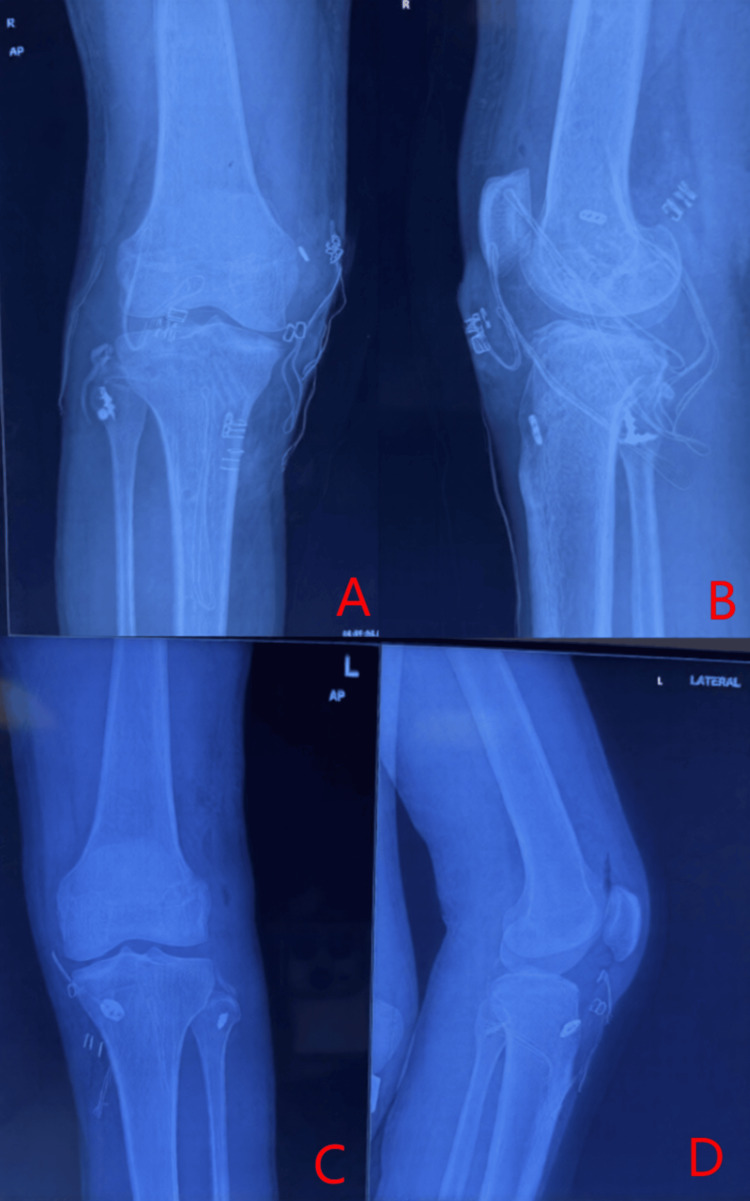
Postoperative X-ray of the right and left knee (A and B) AP and lateral view of the right knee after PLC repair + augmentation and PCL reconstruction. Suture anchor is seen in the fibular head (Larson augmentation), and endobutton is seen on the femoral and tibial side (PCL reconstruction). (C and D) AP and lateral view of the left knee after PLC repair + augmentation and ACL reconstruction. Suture anchor is seen in the fibular head (Larson augmentation), and endobutton is seen on the femoral side with suture disc on the tibial side (ACL reconstruction) AP, antero-posterior; PLC, posterolateral corner; PCL, posterior cruciate ligament; ACL, anterior cruciate ligament

Surgical intervention was planned in two stages, beginning with the right knee. Examination under anesthesia confirmed grade 3 varus and posterior drawer test. An open PLC repair was performed using suture anchors and reinforced with an ipsilateral semitendinosus graft (Larson technique) due to poor tissue quality. The knee was temporarily stabilized with an external fixator to prevent posterior sag and to protect the PLC repair (Figure 4). At six weeks, persistent laxity warranted arthroscopic PCL reconstruction using a peroneus longus autograft with simultaneous lateral meniscus root repair found intraoperatively (Figure 5).

Rehabilitation began five days postoperatively with isometric quadriceps strengthening, range of motion, and patellar mobilization exercises. Partial weight-bearing started at six weeks and progressed to full weight-bearing by eight weeks.

Fifteen days after the first surgery, the left knee underwent arthroscopic ACL reconstruction using a quadruple semitendinosus graft combined with the suture-anchor repair of the PLC avulsion (augmented with gracilis tendon) and the reattachment of the avulsed biceps femoris tendon (Figure 5). Rehabilitation commenced on day 5 with quadriceps and range of motion exercises, followed by progressive mobilization. Partial weight-bearing was allowed at 10 weeks and full activity by 12 weeks.

For the right knee, a two-staged approach was adopted primarily to mitigate the risk of massive fluid extravasation and potential compartment syndrome, as arthroscopic PCL reconstruction requires the extensive elevation of the posterior capsule to visualize and prepare the tibial tunnel for tibial attachment. In the setting of acute multiligamentous injury, preexisting capsular disruptions act as conduits for arthroscopic fluid to escape into the calf compartments. By staging the right side, we allowed for initial capsular and PCL healing and soft tissue stabilization, along with the protection of the PLC repair and augmentation.

At one-year follow-up, the right knee had a functional range of motion from 0° to 120° with grade 1 posterior drawer and stable varus stress. The left knee achieved 0°-140° flexion with grade 1a Lachman and negative pivot shift and varus stress tests. The patient returned to normal daily activities without pain or instability.

## Discussion

Bilateral multiligamentous knee injuries (MLIs) represent an uncommon and severe subset of trauma, observed in less than 5% of all MLI cases. These injuries are usually the result of high-energy mechanisms and are often accompanied by other systemic injuries, including thoracoabdominal and spinal involvement. Consequently, when managing high-impact lower limb trauma, both hips and knees should be carefully examined for associated dislocations or fractures.

Simultaneous bilateral posterolateral corner (PLC) disruptions associated with a single cruciate ligament injury on each side, without frank dislocation, are extremely rare and not well-documented. Management strategies for PLC injuries depend mainly on the timing of surgery, the distinction between avulsion and midsubstance tears, and the overall integrity of the soft tissue envelope. In the present case, both knees demonstrated acute avulsion-type PLC injuries, making early surgical repair a suitable option.

Posterior cruciate ligament (PCL) integrity is routinely assessed using the posterior drawer test. A grade 3 posterior translation exceeding 10 mm strongly indicates combined PCL and PLC disruption, though PLC compromise should also be considered when there is bilateral injury despite a lower-grade posterior drawer.

The dial test provides additional evaluation of rotatory stability, with increased external rotation at 30° alone suggesting an isolated PLC injury and at both 30° and 90° indicating concomitant PCL involvement. However, the reliability of this comparison becomes limited in bilateral cases. Since dial test positivity can also occur in combined medial-sided injuries, differentiation between posterolateral and posteromedial instability is crucial during clinical assessment [7].

A review encompassing 862 knee dislocations identified vascular injury in 18% of cases, emphasizing the need for the prompt evaluation of vascular integrity in such high-energy trauma cases to prevent ischemic limb complications [8]. PLC injuries also demonstrate a notably higher association with peroneal nerve involvement [9], particularly in cases with biceps femoris avulsion, due to varus-induced traction on the nerve [10]. While partial deficits typically recover spontaneously, complete palsy requires surgical management. In this patient, no neural injury was present.

Recent literature demonstrates that a single-stage, combined reconstruction is optimal [11]. However, not many studies have demonstrated the outcome of acute repair, along with the augmentation of the repair using an autograft.

Recent literature has extensively evaluated the outcomes of PLC repair versus reconstruction. Historically, acute avulsion injuries were managed with primary repair; however, studies have shown varying failure rates. Stannard et al. demonstrated that the primary repair of the PLC yielded significantly higher failure rates (20%) compared to reconstruction (4%), suggesting that reconstruction may provide more reliable stability even in the acute setting [12]. Similarly, Levy et al. reported superior outcomes with reconstruction over repair in multi-ligament-injured knees, noting a failure rate of nearly 40% in the repair cohort [13]. Despite these findings, anatomical primary repair may still be considered in carefully selected patients with clean, bony avulsions treated within the first 2-3 weeks of injury, where the soft tissue envelope remains robust [14]. For midsubstance tears or chronic injuries, international expert consensus strongly recommends anatomical reconstruction to restore the normal kinematics of the knee and reduce postoperative instability [15]. Postoperative complications, such as arthrofibrosis, remain a risk in both approaches, underscoring the need for meticulous surgical technique and tailored rehabilitation.

In summary, bilateral PLC injury associated with cruciate ligament disruption is highly unusual. Early recognition, thorough neurovascular assessment, and selective acute repair for avulsion-type injuries can achieve favorable functional results. This case underlines the importance of individualized management and precise surgical timing in complex bilateral MLIs.

## Conclusions

The early repair and selective augmentation of bilateral avulsion-type PLC injuries using semitendinosus and gracilis grafts achieved excellent one-year stability and function. This case reinforces that the prompt, well-indicated augmented repair of acute PLC avulsions can produce outcomes comparable to reconstruction while preserving native tissue and minimizing morbidity. The successful management of this exceedingly rare bilateral presentation underscores the critical need for high clinical suspicion and meticulous physical examination, especially when standard contralateral comparisons are obscured. Furthermore, it highlights that an individualized, staged surgical strategy, paired with a carefully tailored rehabilitation protocol, is essential for optimizing recovery in complex multiligamentous trauma.
